# Metformin Facilitates Osteoblastic Differentiation and M2 Macrophage Polarization by PI3K/AKT/mTOR Pathway in Human Umbilical Cord Mesenchymal Stem Cells

**DOI:** 10.1155/2022/9498876

**Published:** 2022-06-18

**Authors:** Min Shen, Huihui Yu, Yunfeng Jin, Jiahang Mo, Jingni Sui, Xiaohan Qian, Tong Chen

**Affiliations:** ^1^Department of Hematology, Huashan Hospital, Fudan University, Shanghai 200040, China; ^2^Department of Gynecology, Obstetrics & Gynecology Hospital, Fudan University, Shanghai, China

## Abstract

Mesenchymal stem cells (MSCs) are the most promising multipotent stem cells that can differentiate into osteoblasts, chondrocytes, and adipocytes. This cellular flexibility contributes to widespread clinical use of MSCs in tissue repair and regeneration. The immune system is a key player in regulating bone remodeling. In recent years, the association between the immune system and bone metabolism has become an increasing focus of interest. Metformin, a glucose-lowering drug, exerts powerful impact on metabolic signaling. However, whether metformin can modulate bone metabolism or whether metformin can influence immune milieu by regulation of macrophages has not been thoroughly elucidated. Herein, we specifically explored the complex interactions between macrophages and human umbilical cord mesenchymal stem cells (UC-MSCs) in the context of metformin. Our research demonstrated that metformin not only stimulated osteogenesis of UC-MSCs but also influenced the immune system via promoting M2 but reducing M1 macrophages. Mechanically, we found that metformin-treated M2 macrophages possessed more potent osteoinductive capacity in our coculture system. Molecularly, these metformin-stimulated M2 macrophages facilitated osteogenesis via activating the PI3K/AKT/mTOR pathway. As demonstrated by using PI3K-specific inhibitor LY294002, we found that the pathway inhibitor partly reversed osteoinductive activity which was activated by coculture of metformin-treated M2 macrophages. Overall, our novel research illuminated the cooperative and synergistic effects of metformin and M2 macrophages on the dynamic balance of bone metabolism.

## 1. Introduction

Osteoporosis is the most common metabolic bone disease characterized by compromised bone mineral density, deteriorated microarchitecture of trabeculae, increased bone fragility, and hence susceptibility to fractures [1]. An estimated more than 200 million people worldwide suffer from osteoporosis each year [2, 3]. Elderly and postmenopausal women are at the highest risk for developing osteoporosis due to the estrogen deprivation [1, 4, 5]. Glucocorticoid, commonly used in clinical practice, is another most common cause of osteoporosis [6, 7]. Bone resorption by osteoclasts and reconstitution by mesenchymal stem cell- (MSC-) derived osteoblasts are tightly regulated processes of bone homeostasis [8]. Therefore, interventions that promote MSCs towards osteoblast differentiation will be promising alternatives to enhance bone regeneration [9]. Human umbilical cord mesenchymal stem cells (UC-MSCs) have become attractive candidates because the tissues of umbilical cord (UC) are rich sources of MSCs and are easy to collect at a low cost [10, 11].

Metformin, an agonist of the energy sensor AMPK, is currently recommended as the first-line agent for type 2 diabetes mellitus. Apart from its predominant role in lowering high blood glucose, metformin plays crucial roles in a wide variety of biological processes, such as antitumor, anti-inflammatory, antiaging, cardioprotective, and hepatoprotective, and tissue regenerative activities [12–15]. As an old drug with new applications, metformin has been widely documented to regulate metabolism and homeostasis of the bone marrow [14, 16]. It promoted the differentiation and mineralization of induced pluripotent stem cell-derived MSCs and osteoblasts, protected glucose-injured osteoblasts, and even directly involved in the proliferation of osteoblasts [16, 17]. Nonetheless, the underlying effects that metformin exerts on UC-MSCs and immune cells of bone milieu have not yet been thoroughly elucidated.

Macrophages are critical innate immune cells that orchestrate inflammation cascades, immune responses, and tissue repairing [18]. Macrophages have plasticity to adapt to multiple environmental factors and polarize into various functional states [19]. On the one hand, factors such as metformin could polarize the macrophages and inhibit the inflammasome activation [20]. On the other hand, these polarized macrophages could in turn modulate bone milieu and remodeling [21–23]. Therefore, a better understanding of the cross-talk between macrophages and MSCs can revolutionize our understanding of bone homeostasis.

In our present research, we aimed to evaluate the effects of metformin on osteogenesis of UC-MSCs, to investigate the polarization of macrophages in the presence of metformin, and to illuminate the complex regulatory interactions between the immune system and osteogenesis.

## 2. Materials and Methods

### 2.1. The Isolation and Identification of UC-MSCs

The fresh UCs were obtained from healthy young female donors who received normal deliveries at Obstetrics and Gynecology Hospital of Fudan University. Informed written consent was obtained from all donors' families, and tissue collection was approved by the Institutional Review Board of Huashan Hospital of Fudan University. UC-MSCs were isolated and cultured following an established literature procedure [24]. Briefly, Wharton's Jelly was isolated from UCs and then minced into 1-3 mm^3^ fragments under the sterile condition. These pieces were then digested with 2 mg/ml collagenase type II (Sigma-Aldrich, USA) solution for 1 h with agitation (220 rpm) at 37°C. After centrifugation, the cell precipitation was resuspended and filtered through 100 *μ*m cell strainer. Cells were then plated in Dulbecco's modified Eagle's medium (DMEM, Gibco, USA) supplemented with 10% heat-inactivated fetal bovine serum (FBS, Cyagen, China), 1% penicillin/streptomycin (Beyotime, China) in a humidified atmosphere with 5% CO2 at 37°C.

Cells were subcultured at approximately 80% confluence, and passages 3-6 were used for subsequent experiments. The morphological observation was conducted under the phase-contrast microscope (Olympus, Japan). For immunophenotypic identification of UC-MSCs, the surface markers CD44, CD73, CD90, CD45, CD19, CD31, CD34, and CD45 were verified on a flow cytometer (BD FACSCanto II, USA). Cells were stained with APC-conjugated antibodies against human CD44, CD45, CD19, CD31, and CD34, FITC-conjugated antibodies against human CD90, CD105, or PerCP/Cyanine5.5- (PC 5.5-) conjugated antibodies against CD73 (all from BioLegend, USA).

### 2.2. Apoptosis Analysis

Cells apoptosis was detected by Annexin V/Propidium Iodide (PI) double staining kit (BD Biosciences, USA) according to the manufacturer's instructions. After different concentrations of metformin (0, 50, 100, and 200 *μ*M) for 48 h, cells were digested with trypsin, harvested and washed twice with PBS, resuspended in 200 *μ*l binding buffer and labeled with 5 *μ*l Annexin V and 5 *μ*l PI in the dark. The apoptosis distribution was detected with flow cytometer, and the final ratio of apoptosis was calculated as the sum of early and late apoptosis.

### 2.3. CCK-8 Assay

Cell viability of UC-MSCs was estimated with a Cell Counting Kit-8 (CCK-8, Dojindo, Japan). Briefly, cells were seeded into 96-well plate at a concentration of 3 × 10^3^ cells per well. Then, different concentrations of metformin were added to the culture medium. After 48 h of incubation, medium was replaced with 100 *μ*l freshly prepared DMEM containing 10 *μ*l CCK-8 solution. Cells were hatched at 37°C for 2 h in the dark. The absorbance at 450 nm was measured with a spectrophotometer (BioTek SynergyH1, USA). Experiment was performed in triplicate.

### 2.4. Alizarin Red Staining and Alkaline Phosphatase (ALP) Assay

To induce osteogenesis, UC-MSCs were cultured in an osteogenic differentiation media (Cyagen, China) according to the protocols. Briefly, when the confluence rate was about 80%, UC-MSCs were replaced with fresh osteogenic differentiation medium. The medium was changed every 3 days, and cells were continuously cultured for 21 d. The differentiated cells were then fixed in 4% paraformaldehyde for 30 min. After washed twice, cells were ultimately stained with Alizarin Red (Cyagen, China) for 15 min to visualize calcium nodules. As for the ALP enzyme activity, assessment was performed with BCIP/NBT ALP Color Development Kit (Beyotime, China). UC-MSCs were rinsed with PBS twice and fixed by 4% paraformaldehyde after 7 d induction with differentiation media. After fixation, cells were incubated with 1 ml of BCIP/NBT working solution for 30 min in the dark. Color reactions were finally terminated with deionized water. Images were obtained using the phase-contrast microscope.

### 2.5. Real-Time Quantitative PCR (RT-qPCR)

Total RNA was extracted from UC-MSCs using TRIzol reagent (Life Technology, USA) according to the manufacturer's protocol and reversely transcribed into complementary DNA (cDNA) with reverse transcriptase (Takara, Japan). The PCR was preformed using Premix SYBR Green Master Mix (Takara, Japan) on a StepOnePlus RT-PCR machine (Applied Biosystems, USA). Relative mRNA expression levels were calculated using the 2^(−*ΔΔ*CT)^ approach after normalization to GAPDH. Primer sequences of osteogenic genes were listed in Table 1.

### 2.6. Western Blotting

Total protein of UC-MSCs were lysed with RIPA buffer (Beyotime) containing protease and phosphatase inhibitor cocktail (Beyotime). The concentrations were then measured using a BCA kit (Beyotime) according to the protocols. Proteins were separated by SDS-PAGE electrotransferred onto PVDF membranes (Millipore, USA). The membranes were blocked with Quick Blocking Buffer (Beyotime) for 30 min, followed by overnight incubation at 4°C with primary antibodies specific for anti-ALP, RUNX2, OCN, p-mTOR (1 : 1000, Santa Cruz Biotechnology, USA), anti-AKT, p-AKT, mTOR, PI3K, p-PI3K (1 : 1000, CST, USA), and anti-*β*-actin (1 : 1000 Abcam, UK). The membranes were washed and then incubated 1 h with corresponding secondary antibodies. Finally, bands were visualized using an enhanced chemiluminescence under the Image Quant LAS 4000 (GE Healthcare, USA).

### 2.7. Cultivation and Polarization of THP-1

THP-1, the most widely used model for the human monocytes/macrophages, was obtained from Cell Bank of Chinese Academy of Sciences (Shanghai, China), and routinely cultured in RPMI 1640 medium (HyClone, USA) supplemented with 10% FBS and 1% penicillin/streptomycin. We polarized THP-1 monocytes according to the procedure reported previously [25]. THP-1 were firstly differentiated into M0 macrophages with 100 ng/ml phorbol 12-myristate 13-acetate (PAM, Sigma, USA) for 24 h. M0 cells were then polarized into M1 macrophages with incubation with 100 ng/ml LPS (PeproTech, USA) and 20 ng/ml IFN-*γ* (PeproTech) for an additional 48 h. To generate M2 phenotype, M0 populations were exposed to 20 ng/ml IL-4 (PeproTech) and 20 ng/ml IL-13 (PeproTech) for another 48 h. The phenotypes of polarized macrophages were determined by cell makers via flow cytometry. M1 and M2 macrophages were stained using lineage-specific antibodies, with PE anti-human CD86 for M1, APC anti-human CD206 (BioLegend) for M2, and FITC anti-human CD11b (BioLegend) for all monocytes. FlowJo software was used for statistical analysis.

### 2.8. Indirect Coculture of Macrophages with UC-MSCs

A total of 5 × 10^5^ THP-1 macrophages were plated into 0.4 *μ*m pore inserts of 6-well Transwell plates (Corning, USA) in 2 ml of medium. M1, M2, or metformin-treated M2 macrophages were induced and cultured in the luminal chamber using the polarization methods above. To induce metformin-treated M2 macrophages, different concentrations of metformin (0, 50, and 100 *μ*M) were treated during the process of M2 polarization. After the polarization, the inducing medium was changed with regular RPMI 1640 medium, and the inserts were added to the abluminal chamber containing UC-MSCs at 50% confluence. Total RNA and proteins were isolated from UC-MSCs after 48 h cell-cell indirect coculture.

### 2.9. Immunofluorescence

The UC-MSCs were attached to glass slides. After 48 h coculture with M2 or metformin-treated M2 macrophages (metformin 100 *μ*M), UC-MSCs were fixed with 4% paraformaldehyde for 15 min, permeabilized by 0.3% Triton X-100 for 30 min, and blocked in 1% BSA for another 30 min. Cells were then incubated overnight at 4°C with monoclonal antibodies against p-mTOR (1 : 100, Santa Cruz Biotechnology). After washing three times, slides were incubated with Alexa Fluor 488-conjugated secondary antibody for 1 h and then labeled cell actin filaments with Alexa Fluor 555-conjugated phalloidin (Beyotime) for 20 min. Nuclei were counterstained with DAPI. Fluorescence images were captured using a Leica TCS SP8 confocal microscope (Leica Microsystems, Germany).

### 2.10. Statistical Analysis

Data obtained from three independent experiments were presented as means ± SD. All statistical analyses were performed using GraphPad Prism 9.0 (GraphPad Software, CA). The two-tailed Student's *t*-test were used to compare two groups of data. One-way analysis of variance was assessed for the comparison of differences between three or more groups. *P* values < 0.05 were defined as the significance threshold.

## 3. Results

### 3.1. The Identification and Characterization of UC-MSCs

**Figure 1:**
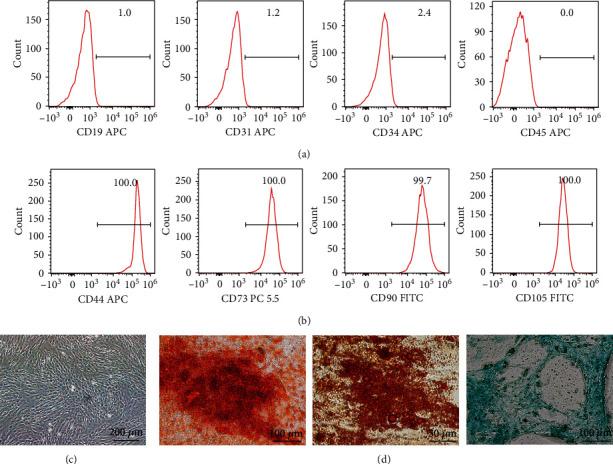
The characterization of UC-MSCs. (a, b) The identification of negative surface makers CD19, CD31, CD34, and CD45 and positive makers CD44, CD73, CD90, and CD105 of UC-MSCs from passage 5 using flow cytometry. (c) Morphological image under an inverted microscope. Scar bar = 200 *μ*m. (d) Osteoblastic ability was validated by Alizarin Red staining 21 d after differentiation. Scar bar = 100 *μ*m; adipogenesis was assessed with Oil Red O staining 14 d after inducing. Scar bar = 50 *μ*m; chondrogenic differentiation was stained with Alcian Blue dye. Scar bar = 100 *μ*m.

UC is one of the most important sources of MSCs. In this study, we isolated human UC-MSCs and identified cells with established minimum but widely accepted criteria [11, 26]. The immunophenotype results with flow cytometry showed negative for a cluster of surface makers CD19, CD31, CD34, and CD45 (Figure 1(a)), while strongly positive for CD44, CD73, CD90, and CD105 (Figure 1(b)). The plastic-adherent ability and spindle-like morphology with self-renewal capability were confirmed by optical images (Figure 1(c)). Their capacity to differentiate into multiple lineages was assessed by osteogenic, adipogenic, and chondrogenic capacities (Figure 1(d)). Calcium deposition of osteogenically differentiated cells was confirmed with Alizarin Red staining, the formation of small cytoplasmic lipid droplets was detected by Oil Red O dye, and acidic mucopolysaccharide was visualized with Alcian Blue solution. The above identification confirmed our successful isolation of UC-MSCs.

### 3.2. Metformin Did Not Affect the Growth of UC-MSCs In Vitro

**Figure 2:**
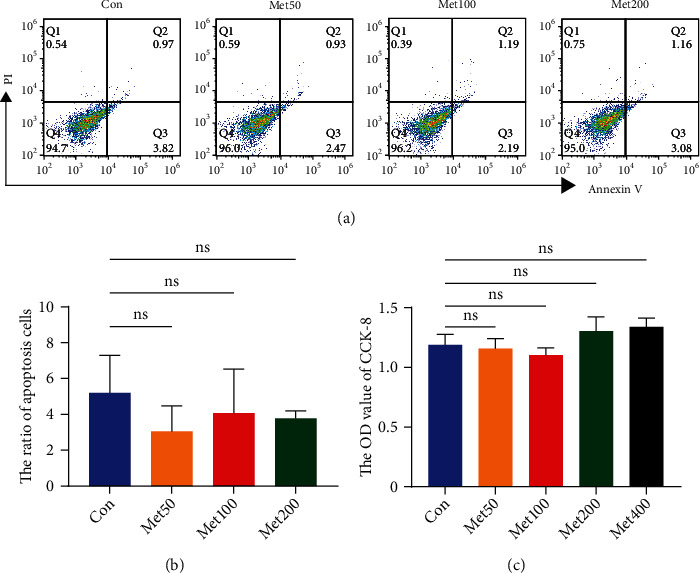
Metformin did not influence the proliferation of UC-MSCs in vitro. (a, b) Representative graphs and statistical analysis of Annexin V/PI double staining were displayed. (c) The OD value of CCK-8 after different concentrations of metformin (0, 50, 100, 200, and 400 *μ*M) for 48 h. Data were expressed as mean ± SD from three independent experiments. Met: metformin; ns: no significance.

To investigate the effects of metformin on UC-MSCs growth, we measured apoptosis and cell viability after treatment with different concentrations of metformin for 48 h. As shown in Figures 2(a) and 2(b), metformin with concentrations of 0, 50, 100, and 200 *μ*M did not show proapoptotic effect on UC-MSCs. Moreover, we adopted the CCK-8 assay to evaluate cytotoxic effects with different doses of metformin as indicated. The results exhibited neither growth inhibiting nor promoting effects, even in relatively high dose of the 400 *μ*M group (Figure 2(c)). Thus, we considered that metformin had no signs of cytotoxicity and influence on proliferation for UC-MSCs within our tested dose ranges.

### 3.3. Metformin Promoted UC-MSCs towards Osteoblastic Differentiation

**Figure 3:**
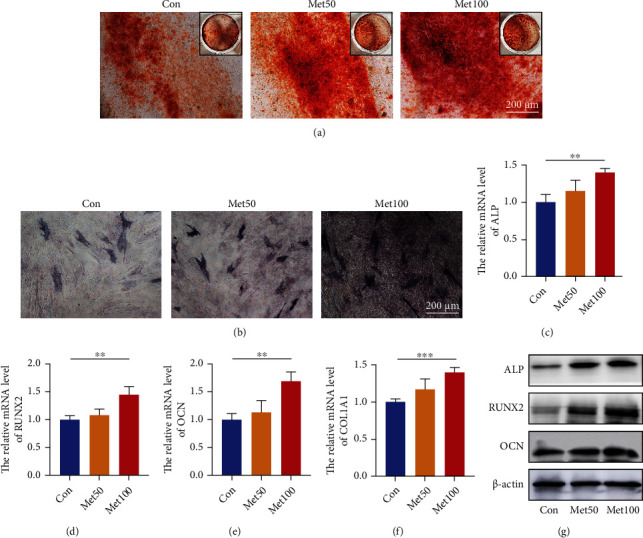
Metformin enhanced osteogenic differentiation capacity of UC-MSCs. (a) Osteogenically differentiated cells were identified with Alizarin Red staining after 21 days incubation. Scar bar = 200 *μ*m (100x). (b) ALP activity of UC-MSCs was evaluated after 7 days incubation. Scar bar = 200 *μ*m (100x). (c–f) Quantitative analysis of osteogenic genes expression by RT-qPCR, including ALP (c), RUNX2 (d), OCN (e), and COL 1A1 (f). *n* = 3; ^∗∗^*P* < 0.01, ^∗∗∗^*P* < 0.001. (g) Osteogenic proteins of ALP, RUNX2, and OCN were assessed by western blot.

To elucidate the underlying effect of metformin on UC-MSCs, osteogenic differentiation and mineralization were examined. We firstly detected the osteogenically differentiated cells with Alizarin Red staining. We stimulated UC-MSCs with different concentrations of metformin (0, 50, and 100 *μ*M) in the presence of osteogenic induction medium for 21 d. Calcium deposition was visualized in deep red and increased in a concentration-dependent manner (Figure 3(a)). To further confirm the promotive effects on osteogenesis, we conducted ALP assay and results showed greater enzyme activity for ALP in the higher dose group (Figure 3(b)). Furthermore, RT-qPCR were used to confirm the expression of osteoblastic markers. As shown in Figures 3(c)–3(f), osteogenic gene expression of ALP, RUNX2, OCN, and COL1A1 in the group with 100 *μ*M metformin were significantly increased compared with the control. As expected, our western blot bands showed higher expression of osteogenic makers in metformin-treated group, including ALP, RUNX2, and OCN (Figure 3(g)). Overall, these results confirmed that metformin had an osteogenesis-promoting effect on UC-MSCs.

### 3.4. Metformin Modulated Macrophage Polarization by Inducing M1/M2 Switching

**Figure 4:**
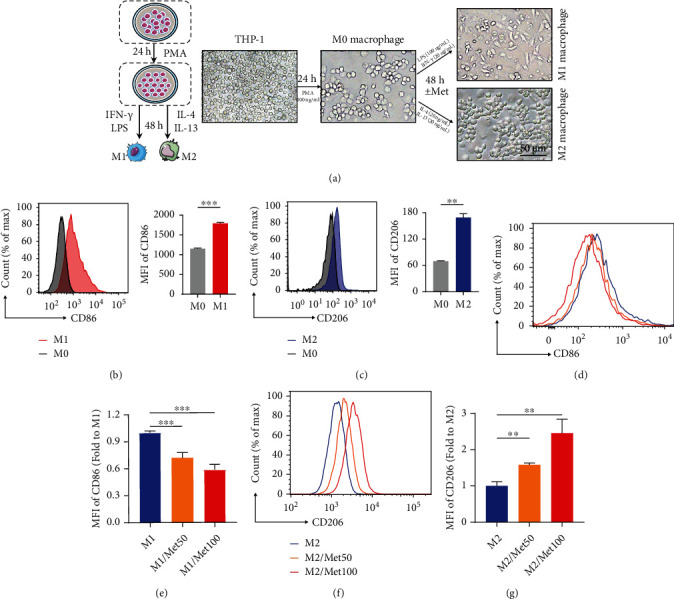
Metformin regulated macrophage polarization towards M2 phenotype. (a) Schematic diagram of polarization of macrophages from THP-1 to M1/M2 populations. (b, c) Representative graphs and statistical histograms of MFI of CD86 maker in M1-induced group and CD206 in M2-induced group versus M0 populations. (d) Flow cytometry plot of CD86 (M1 maker) with different doses of metformin. (e) Quantitative analysis of the MFI of CD86. (f) Representative image of CD206 (M2 maker) with different doses of metformin. (g) Quantitative analysis of the MFI of CD206. Data represented as mean ± SD from three independent experiments. ^∗∗^*P* < 0.01, ^∗∗∗^*P* < 0.001.

Given that metformin is a key inflammatory modulator, we next aimed to investigate whether metformin had a substantial impact on immune modulation especially for macrophages. Thus, we induced the polarization of macrophages in vitro. THP-1 cells were firstly incubated with PAM for 24 h to obtain M0 phenotype. Subsequently, IFN-*γ* and LPS were used for M1 polarization, or IL-4 and IL-13 for M2 phenotype (Figure 4(a)). The morphological images of different state macrophages were displayed in our Figure 4(a). Our successful polarization of M1 and M2 phenotypes were identified with CD86 and CD206 markers, respectively, via flow cytometry. Our data demonstrated CD86 was remarkably increased in M1 macrophages (Figure 4(b)) while CD206 sharply higher in M2-induced group (Figure 4(c)). During process of 48 h polarization, different concentrations of metformin (0, 50, and 100 *μ*M) were added. As was visually and statistically displayed in Figures 4(d) and 4(e), combined induction with metformin elicited a significant decrease of CD86 (M1 phenotype) expression, while a marked increase in CD206 (M2 maker) (Figures 4(f) and 4(g)). These data suggested that metformin could modulate the phenotypes of M1 and M2 macrophages and that the phenotypic transition seemed to favor M2 polarization.

### 3.5. Coculture of Macrophages with UC-MSCs Influenced Osteogenesis Capacity

**Figure 5:**
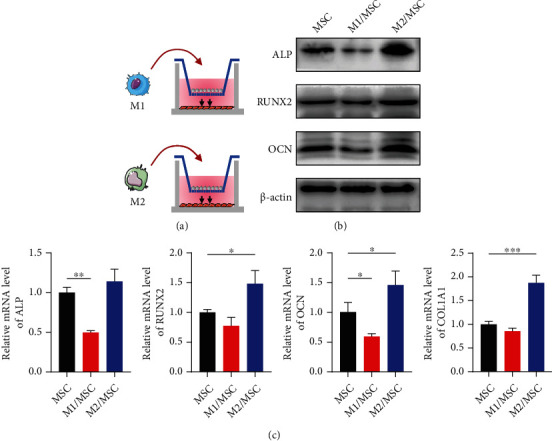
Indirect coculture of macrophages with UC-MSCs altered osteoblastic differentiation ability. (a) Schematic graphs for M1 and M2 macrophages cocultured with UC-MSCs in Transwell plates (membrane pore = 0.4 *μ*m). (b) Detection of osteogenic-related proteins ALP, RUNX2, and OCN by western blot. (c) Quantitative analysis of osteogenic genes ALP, RUNX2, OCN, and COL1A1 by RT-qPCR. Data are presented as mean ± SD from triplicate experiments. ^∗^*P* < 0.05, ^∗∗^*P* < 0.01, and ^∗∗∗^*P* < 0.001.

Molecules in surrounding milieu, to a large extent, changed the polarization states of macrophages [27]. In turn, these polarized macrophages can exert critical roles in regulating microenvironmental elements including stroma cells [28]. Thus, in the present study, we were to explicitly explore the roles and interactions of macrophages with UC-MSCs. We firstly polarized M1 and M2 macrophages, respectively, in the upper chamber of a Transwell system and then cocultured them with UC-MSCs in the lower chamber (Figure 5(a)). Osteogenic markers were assessed after 48 h coculture in the lower chamber. As shown in Figure 5(b), osteogenic proteins, including ALP, RUNX2, and OCN, were significantly enriched in the M2/MSC-cocultured group compared with the MSC alone or M1/MSC-cocultured group. In addition, M2 macrophages suggested a promotive effect on osteogenic genes (ALP, RUNX2, OCN, and COL1A1) by RT-qPCR, while the M1/MSC-cocultured group indicated a downward trend (Figure 5(c)). Prior studies suggested M2 macrophages secreted osteogenic factors to promote osseointegration [29]. Consistently, our study indicated THP-1-derived M2 macrophages enhanced osteogenesis of UC-MSCs whereas M1 macrophages might act in the opposite fashion.

### 3.6. Metformin-Treated M2 Macrophages Promoted Osteogenesis by PI3K/AKT/mTOR Pathway

**Figure 6:**
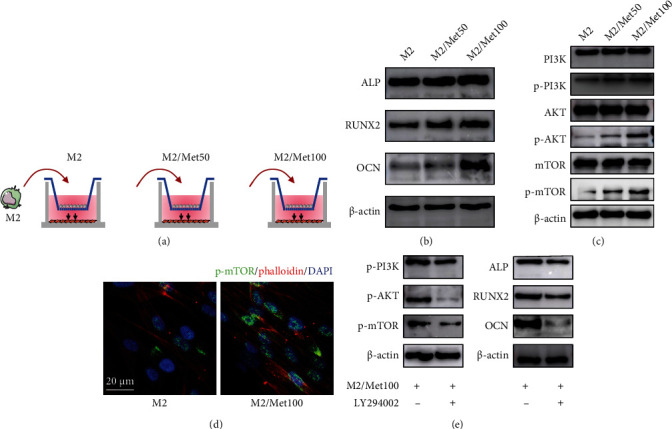
Metformin-pretreated M2 macrophages enhanced osteoblastic differentiation via the PI3K/AKT/mTOR pathway (a) The diagram of metformin (0, 50, and 100 *μ*M)-treated M2 macrophages cocultured with UC-MSCs. (b) Expression levels of osteogenic-related proteins (ALP, RUNX2, and OCN) after coculture with metformin-treated M2 macrophages for 48 h. (c) Expression levels of PI3K/AKT/mTOR-pathway-related proteins (PI3K, p-PI3K, AKT, p-AKT, mTOR, p-mTOR). (d) Representative immunofluorescence staining of p-mTOR in M2-cocultured or M2/Met100-cocultured UC-MSCs. p-mTOR (green), phalloidin (red), and counterstained with DAPI (blue). Scar bar = 20 *μ*m. (e) Expression levels of p-PI3K, p-AKT, p-mTOR, and ALP, RUNX2, and OCN after treatment with or without LY294002 (50 *μ*M).

It has been documented that macrophage carries out a wide range of functions by directly acting on MSC [30]. However, whether metformin and M2 macrophages could exert synergistic effects on osteogenesis is still unknown. We pretreated M2 macrophages with different concentrations of metformin (0, 50, and 100 *μ*M), then cocultured them indirectly with UC-MSCs for 48 h, and investigated the influence on osteogenic ability (Figure 6(a)). Our western blot results revealed that metformin-stimulated M2 macrophages possessed more potent osteogenic ability as indicated by higher levels of ALP, RUNX2, and OCN, when compared with the M2 group (Figure 6(b)). Given that metformin facilitated M2 macrophages, we therefore concluded that interventions promoting M2 polarization boosted osteogenesis. The PI3K/AKT/mTOR pathway is a key link between inflammation and bone formation [31, 32]. We focused on the expression levels of p-PI3K, p-AKT, and p-mTOR by western blot, and they were markedly increased after coculture with metformin-treated M2 macrophages (Figure 6(c)). Our conclusion was further verified by higher expression of p-mTOR in immunofluorescence (Figure 6(d)). To confirm the involvement of the PI3K/AKT/mTOR signaling, we blocked upstream target of this signaling with PI3K-specific inhibitor LY294002. Our western blot results showed that inhibitor LY294002 suppressed the PI3K/AKT/mTOR pathway, as assessed by lower expression of p-PI3K, p-AKT, and p-mTOR (Figure 6(e)). Concurrently, osteogenesis proteins ALP, RUNX2, and OCN were decreased (Figure 6(e)). Overall, these results demonstrated that intervention with metformin not only promoted M2 polarization but also collaborated with M2 macrophages to induce osteogenic differentiation through activating the PI3K/AKT/mTOR pathway.

## 4. Discussion

In this study, we determined whether metformin could play a crucial role in osteogenesis in the context of macrophages and its underlying mechanisms. Our research demonstrated that metformin promoted osteogenesis. Moreover, we concluded that metformin played a regulatory role in switching M1 to M2 macrophages, which facilitated a transition from inflammation to tissue regeneration state. Subsequently, we investigated the effects of metformin-treated M2 macrophages and finally revealed that metformin-stimulated M2 macrophages regulated the osteoblastic differentiation by the PI3K/AKT/mTOR pathway.

Osteoporosis is a globally prevalent public health problem characterized by low bone mass and microarchitectural deterioration, predisposing a person to high risk of fragility fractures [4, 33]. Postmenopausal osteoporosis is a primary contributor to osteoporosis, with overwhelmingly higher prevalence in postmenopausal elder women [1, 2, 4, 33]. Secondary osteoporosis such as glucocorticoid-induced osteoporosis may also underestimate fracture risks [34, 35]. Despite the tremendous progress of antiosteoporosis regimens, it is still inadequate especially under the condition that the bone immune microenvironment is uncertain and unrecognized [34, 36]. Thus, in our study, we detected osteoinductive agents of UC-MSCs with metformin and explored their complex interactions with macrophages.

MSCs are the most extensively studied stem cells and intensely investigated for clinical translation within the last decades. As multipotent stem cells, MSCs are used to treat bone diseases because they can differentiate into osteoblast to comply bone formation and repairing [11]. Recent studies have reported that aberrant differentiation of MSCs was closely associated with several pathophysiologic processes including the disrupted adipoosteogenic balance [37]. Investigations have demonstrated that factors inducing adipocytes inhibited osteogenesis, and conversely bone-inducing factors hindered adipogenesis, dictating the reciprocal regulation between adipocytes and osteoblasts [37, 38]. Thus, we speculate external interventions that direct MSCs downwards osteoblast lineage are the promising therapies for osteoporosis. In this research, we confirmed that metformin, with no signs of cytotoxicity in vitro, enhanced bone-forming potential of UC-MSCs.

A highly complex immune system involves in interactions with many immune cells which can produce various inflammation-related cytokines changing bone microstructure. Among these cells, macrophages possess a significant role in immunoregulation and bone homeostasis [39]. Macrophages exist in two main polarization states, classically activated macrophages (M1) and alternatively activated macrophages (M2). To better understand the interactions between innate immunity and bone remodeling, we explored the influence on macrophages with chemical treatment of metformin. Previous studies have demonstrated that metformin ameliorated inflammation in vivo and polarized mouse RAW264.7 macrophages into M2 phenotype in vitro [20, 40]. Consistently, we revealed that metformin could effectively switch human THP-1-polarized macrophages from M1 into M2 phenotype.

It is widely believed that different macrophage polarization states contribute to different conditions of bone metabolism [3, 21, 22]. Zhou et al. demonstrated that induction of M2 polarization facilitated osteogenic differentiation [21]. Kang et al. demonstrated that the M2 macrophage extracellular vesicles increased osteoinductive gene expression of MSCs [41]. Similar results were observed in our coculture system. We revealed that THP-1-derived M2 macrophages promoted bone-forming potential of MSCs while M1 decreased. Recently, Qing et al. reported that metformin-induced M2 macrophages accelerated the wound healing via regulating AMPK/mTOR/NLRP3 inflammasome singling [20]. Jing et al. showed that metformin-polarized macrophages relieved obesity-associated inflammation state [40]. However, whether metformin-stimulated macrophages could guide MSCs downwards the osteogenic lineage remains a mystery. In our experiments, different concentrations of metformin costimulated with IL-4 and IL-13 during polarization of M2 macrophages. After 48 h incubation, metformin-treated M2 groups showed more potent bone-forming capacity, which suggested that metformin treatments were multifunctional and more effective strategies by targeting both MSCs and macrophages.

PI3K/AKT/mTOR is a complex and important signaling with multiple regulators and effectors. These effectors, including insulin, glucose, and growth factors and cytokines, can initiate PI3K/AKT/mTOR signaling [31]. PI3K/AKT/mTOR fulfills functions in many cellular processes essential for homeostasis, including cell cycle, proliferation, autophagy, inflammation, and metabolism [31, 42]. Moreover, increasing evidence supports the involvement of PI3K/AKT/mTOR in bone metabolism and remodeling [43–45]. Namely, Liu et al. proved that morroniside promoted the osteogenesis through PI3K/Akt/mTOR signaling [43]. Ma et al. reported that hydrogen sulphide promoted osteoclastogenesis by inhibiting the PI3K/AKT/mTOR pathway [44]. However, Tanaka and coworkers supported that suppression of the AKT/mTOR signal pathway enhanced dentinogenic capacity of stem cells from apical papilla [45]. Thus, we sought to determine the transcriptional levels of the PI3K/AKT/mTOR pathway after coculture of UC-MSCs with metformin-treated M2 macrophages. Consistent with majority of findings, our coculture results revealed increasing osteoblastic ability with the activated PI3K/AKT/mTOR pathway. Moreover, this osteoblastic activity was partially reversed by the pharmacological inhibitor of PI3K. Overall, we verified that metformin-enhanced M2 macrophages facilitated osteogenesis of UC-MSCs via activation of PI3K/Akt/mTOR signaling.

## 5. Conclusion

Taken together, our research provided evidence that the application of metformin could enhance osteogenic differentiation of UC-MSCs without no signs of cytotoxicity in vitro. In addition, metformin-treated macrophages showed an increase in M2 marker while a descending tendency for M1 phenotype. Besides, our findings revealed that metformin-treated M2 macrophages were more competent to promote osteogenesis via activating PI3K/AKT/mTOR signaling. In summary, this novel research provided an overview that the immune modulation of macrophages with metformin was in responsible for the positive regulations of osteogenesis.

## Figures and Tables

**Table 1:** Primers used in RT-qPCR.

Gene	Primer	Sequence
ALP	Forward	CTGGTACTCAGACAACGAGATG
	Reverse	GTCAATGTCCCTGATGTTATGC
RUNX2	Forward	AAGCTTGATGACTCTAAACC
	Reverse	TCTGTAATCTGACTCTGTCC
OCN	Forward	GGCGCTACCTGTATCAATGG
	Reverse	GTGGTCAGCCAACTCGTCA
COL1A1	Forward	AAAGATGGACTCAACGGTCTC
	Reverse	CATCGTGAGCCTTCTCTTGAG
GAPDH	Forward	CAGGAGGCATTGCTGATGAT
	Reverse	GAAGGCTGGGGCTCATTT

## Data Availability

The data used to support the findings of this study are available from the corresponding author upon request.
